# Improving vaccination uptake in pediatric Cochlear implant recipients

**DOI:** 10.1186/s40463-018-0308-5

**Published:** 2018-09-17

**Authors:** Lisa Jin, Paula Téllez, Ruth Chia, Daphne Lu, Neil K. Chadha, Julie Pauwels, Simon Dobson, Hazim Al Eid, Frederick K. Kozak

**Affiliations:** 1 Faculty of Medicine, University of British Columbia, 317-2194 Health Sciences Mall, Vancouver, BC V6T 1Z3 Canada; 2 Division of Pediatric Otolaryngology-Head and Neck Surgery, BC Children’s Hospital, 4480 Oak St, Vancouver, BC V6H 3N1 Canada; 3 Department of Audiology, BC Children’s Hospital, 4480 Oak St, Vancouver, BC V6H 3N1 Canada; 4 Sidra Medical and Research Centre, Doha, Qatar; 5 Department of Surgery, Division of Otolaryngology King Fahad Specialist Hospital, Dammam, Kingdom of Saudi Arabia

**Keywords:** Cochlear implant, Meningitis, Otolaryngology, Preventative medicine, Paediatrics, Public health, Vaccination

## Abstract

**Background:**

An Infectious Disease vaccine specialist joined our institution’s Cochlear Implant Team in 2010 in order to address the high percentage of non-compliance to immunization prior to surgery identified previously from an internal review. The purpose of this study was to (1) review the immunization status of cochlear implant recipients in 2010–2014, (2) assess if introducing a vaccine specialist made a significant change in vaccination compliance and (3) elucidate any barriers to vaccination compliance.

**Methods:**

Retrospective chart review and a telephone survey. Medical records of 116 cochlear implant recipients between 2010 and 2014 were reviewed. A telephone survey was conducted to obtain the current vaccination status in children who required post-operative vaccinations with incomplete records on chart review and, if applicable, the reason for non-compliance.

**Results:**

Between 2010 and 2014, 98% of children were up-to-date at the time of surgery, compared to 67% up-to-date at the time of surgery between 2002 and 2007. 27 children were included in our post-operative immunization analysis. 29.6% (8/27) failed to receive necessary vaccinations post-surgery. Pneumovax-23, a vaccine for high-risk patients (such as cochlear implant candidates) was missed in all cases.

**Conclusion:**

Pre-operative vaccination for cochlear implant recipients improved dramatically with the addition of a vaccine specialist. However, a significant proportion of patients requiring vaccinations post-surgery did not receive them. The main reason for non-compliance was due to parents being unaware that their children required this vaccine postoperatively by being “high-risk”.

Although improvement was demonstrated, a communication gap continued to impede the adequacy of vaccination uptake in pediatric cochlear implant recipients following surgery at age 2 when the high-risk vaccine was due.

## Background

Advances in technology over the last few decades have greatly impacted patient care and quality of life; cochlear implants prove to be an excellent example. A cochlear implant (CI) is a 2-part electrode with an external microphone and an internal electrode implanted in the cochlea that provides direct electric stimulation to the auditory nerve fibers [1, 2]. CIs allow those with profound sensorineural hearing loss to appreciate hearing and to develop the ability to communicate through spoken language [1, 2]. However, as with any invasive intervention, there lies the risk of infection. Due to the close proximity of the cochlea to the brain, post-operative bacterial pneumococcal meningitis is and has been a significant concern for CI surgeons and recipients.

The incidence of *Streptococcus pneumoniae* meningitis in children with CIs has been reported to be 16 to 30 times higher than the general population due to multiple predisposing risk factors. Cochleovestibular malformation, a major risk factor for pneumococcal meningitis, is a common cause for children with sensorineural hearing loss. Electrode insertion, failure to seal the cochleostomy, and the lack of appropriate meningitis vaccines strictly for high-risk populations are additional identified risk factors [1, 3, 4]. Moreover, a much lower inoculation threshold of *S. pneumoniae* is required to induce meningitis through the cochlea compared to other means of entry [5]. Although many of these factors are beyond control, ensuring these children are properly immunized against the highly virulent *S. pneumoniae* subtypes before and after surgery is critical in harm-reduction for the CI population. Currently CI recipients in our province follow the provincial high-risk vaccination schedule for pneumococcal meningitis prevention. Under the high-risk schedule, CI patients receive 2 additional vaccines, an additional dose of 13-valent pneumococcal conjugate vaccine (PCV-13) at 6 months and the 23-valent pneumococcal polysaccharide vaccine (PPV-23) at 24 months of age, for greater protection against *S. pneumoniae*.

**Fig. 1:**
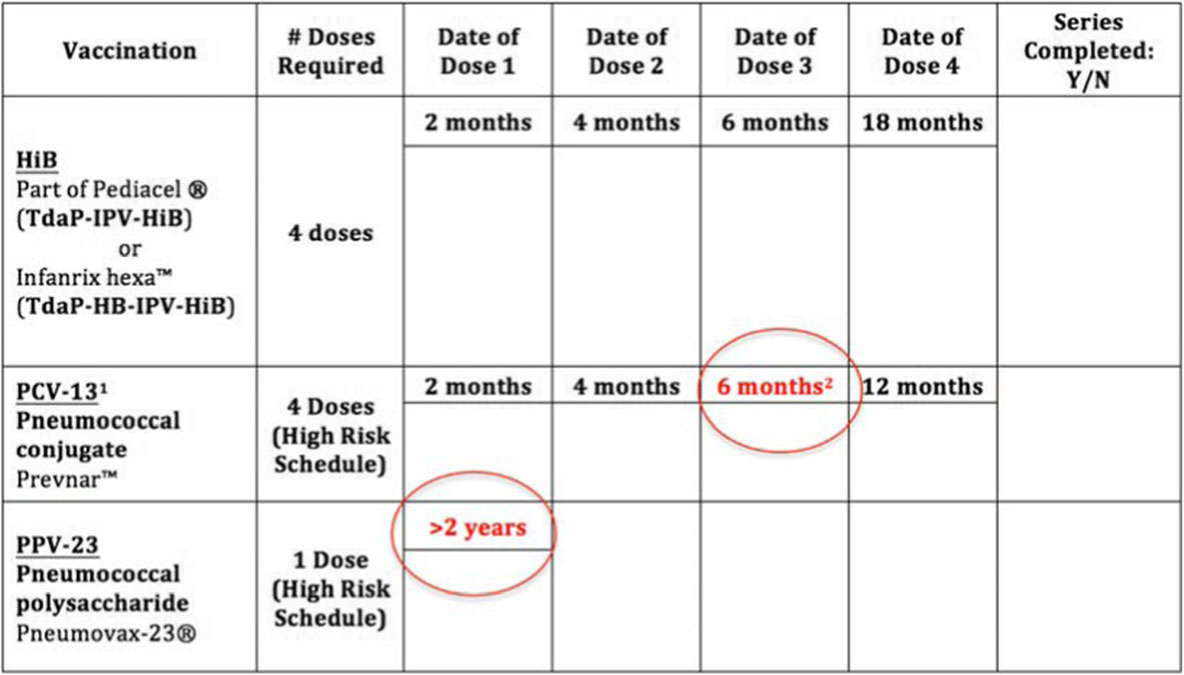
Vaccination schedule for children at high risk for meningitis (ie – Cochlear Implant Recipients). Additional high-risk vaccine doses are circled and outlined in red text. ^1^Pneumococcal conjugate (PCV-13) is required for children under the age of 5. ^2^The high-risk schedule for PCV-13 series only applies to patients < 1 years of age at the time of candidacy assessment. If a child is over the age of 1 at the time of cochlear implant candidacy assessment, only 3 doses are required

A previous internal review of vaccination rates in pediatric CI patients at our institution, implanted between 2002 and 2007 [unpublished], revealed that 33% of patients were not up-to-date with their meningitis vaccinations at the time of their surgery. Reported barriers to vaccination compliance included confusion from changes in provincial vaccination schedules, the language barrier associated with the province’s high immigration rate, difference in vaccination requirements between provinces and lack of communication between patients’ families and health providers. Recognizing that inadequate vaccination of CI patients largely stemmed from confusion and lack of communication over the high-risk schedule requirements, in 2008 the Cochlear Implant Team at our hospital partnered with a vaccine specialist to address this significant concern. As a result, a structured plan was put into place utilizing a preoperative template with both the routine and high risk vaccine schedules clearly outlined to be confirmed by either the vaccine infectious disease specialist or the cochlear implant surgeon (Fig. 1). Any confusion surrounding the vaccine status of the patient was reviewed with the vaccine specialist. Situations where implant candidates either came from a different province or another country where in both instances vaccine schedules differ were closely reviewed and modifications made to comply with the province’s standards. In order to improve the confusion and lack of adequate vaccination after the internal review, our institution created a policy that required patients to be up-to-date with their pneumococcal meningitis vaccinations prior to surgery. In some instances this may result in a delay in implantation. The option to vaccinate patients those vaccines that are missing on the day of surgery is not an optimal scenario as it takes between two to eight weeks depending on the vaccine to achieve adequate immune response to the vaccine and obtain maximal immunity. Although there is no firm data to support this stance that is taken by our program the above reasoning is why this position has been taken. The resultant delay is felt not to be significant enough to affect the long term outcome of the patient’s cochlear implantation.

This study aimed to i) review the current immunization status of recently implanted patients at our institution since the aforementioned change was made, ii) assess if this change made a significant impact on the number of patients with inadequate vaccinations and iii) elucidate any barriers that continue to exist in vaccination compliance pre- and post-CI surgery. Of note, the high-risk immunizations and vaccinations referred in this review are specific to those for pneumococcal meningitis.

## Methods

The objectives of this study were to review the vaccination status of CI patients implanted between 2010 and 2014, compare findings with the previous internal review (2002–2007) and identify barriers to vaccination compliance. Ethical approval was obtained by the Institutional Ethics Board and informed verbal or written consent was obtained from the parents and/or legal guardians of children who participated in the telephone interview.

The study consisted of two parts:

1. *Chart Review:* A retrospective Chart Review was conducted to determine the vaccination status for all CI recipients operated on at our institution between 2010 and 2014 i) at the initial candidacy assessment, ii) during surgery and iii) post-operatively, if appropriate. Post-operative vaccination status was only collected if patients required additional meningitis immunizations after surgery. These include children that were operated on with missing vaccinations and children who were not old enough at the time of surgery to have completed the high-risk meningitis vaccination series (due at 24 months of age). For children who received two CIs during the study period at different times, the vaccination status (at candidacy assessment, during surgery and post-operatively) for their first CI was collected.
2. *Telephone Survey*: All children identified with missing vaccination information on post-implant vaccinations that could not be found on the chart review were contacted for a telephone survey. Information was collected regarding current vaccination status and the reason for non-compliance, if applicable.

### Chart review

The study population included all children (19 years or younger at time of their procedure) who had received a cochlear implant at our institution between January 1, 2010 and December 31, 2014, inclusive. Re-implanted recipients were excluded from the study. A total of 116 children met the study criteria. Hospital and clinic records were reviewed for basic demographics (gender, place of birth, date of immigration), clinical history of hearing loss, records of meningitis vaccination series (*Haemophilus influenzae* type 2, 13-valent pneumococcal conjugate vaccine, 23-valent pneumococcal polysaccharide vaccine), and focused pre-surgical, surgical and post-surgical details.

Once children are considered candidates for CI at our institution, they are required to follow the high-risk meningitis vaccination schedule thereafter. Children with a CI received prior to the study period are considered high-risk for pneumococcal meningitis and thus are required to follow the high-risk schedule during the initial candidacy assessment for their second CI. The high-risk meningitis vaccination schedule includes four doses of *Haemophilus influenzae* type 2 vaccine (HiB), four doses of 13-valent pneumococcal conjugate vaccine (PCV-13) and a single dose of 23-valent pneumococcal polysaccharide vaccine (PPV-23) for children over the age of 2 who have completed the PCV-13 series. Figure 1 shows the additional doses required in the high-risk schedule. There are two exceptions to this schedule: i) a fourth PCV-13 dose is not required for children identified as a CI candidate after the age of 1 and ii) PCV-13 is recommended for children under the age of 5 only. Since the PCV-13 vaccine became part of the standard immunization series in 2000, PCV-13 records of children born prior to 2000 were not reviewed as they are over the age of 5 during the study period (2010–2014).

A child was deemed “up-to-date” with his or her vaccinations at the time of assessment with the cochlear implant team if all immunization series were completed or if the most recent age-appropriate dose of each immunization series was received at the time of this visit. This definition was also applied in the assessment of vaccination status at the time of surgery.

For children who were not “up-to-date” with their vaccinations, individualized immunization catch-up programs were implemented to ensure the most appropriate vaccines are received prior to surgery. A vaccine infectious disease specialist was consulted to determine whether or not these individuals were up-to-date with their vaccinations at the time of surgery on a case-by-case basis. The catch up vaccines prior to surgery were given by either the local public health unit, by the patient’s family practitioner, or on rare occasions, by the vaccine infectious disease specialist.

### Telephone survey

A telephone survey was administered to the parent and/or legal guardian of cochlear implant recipients with missing vaccination information after the chart review. These include children who were missing vaccines at the time of surgery or children who were not old enough to complete the immunization series prior to surgery and records were not updated in the chart during the review. A record of any additional vaccines (HiB, PCV-13 and PPV-23) received after surgery was recorded and if applicable, reasons for vaccine non-compliance were documented. Parents were made aware of any vaccine their child was missing. A letter outlining the vaccines each child was missing was also sent out to these families to be taken into Public Health or their family physician for appropriate vaccination catch-up. Public Health Records were also reviewed with permission.

### Statistical analysis

Descriptive statistics including means, ranges and standard deviation were used to summarize continuous variables. Categorical variables were summarized with percentages. Absolute percentage change and odds ratio were used to compare results from this study and the 2008 internal review.

## Results

One hundred sixteen children received cochlear implants at our institution between 2010 and 2014. The age range of the children was less than 1 year of age to 19 years (median 5.72 years; SD 4.77 years). The male to female ratio was 1.2:1 (63:53).

Of these 116 children, 37 had a cochlear implant prior to the study period and received a second CI between 2010 and 2014. These children were considered high-risk patients at the time of candidacy assessment for the second implant. From the remaining 79 patients, 48 received one CI and 31 received two CIs during the study period. Twelve children received both implants at the same surgery (bilateral simultaneous CI) and 19 children underwent two separate cochlear implant surgeries (bilateral sequential CI).

### Vaccination status at time of candidacy assessment with the CI team

**Fig. 2:**
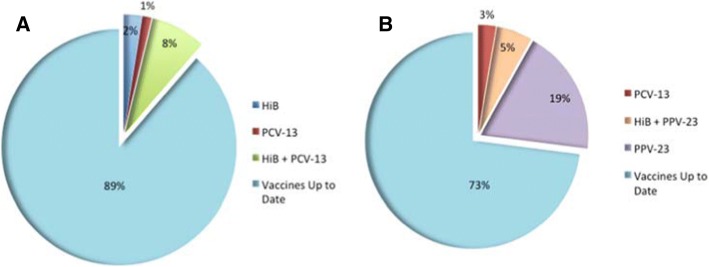
Vaccines missing at time of candidacy assessment with the Cochlear Implant team. **a**–First time CI recipients. **b**–Patients receiving their second CI. Vaccines up to date – either the individual has completed all vaccinations or has received the most recent age-appropriate vaccination. HiB – Can be part of Pediacel ® or Infranrix Hexa depending on the age. PCV-13 – Pneumococcal Conjugate (Prevnar™). PPV-23 – Pneumococcal Polysaccharide (Pneumovax-23 ®)

A total of 19/116 patients (16%) were not up-to-date at the time of candidacy assessment (Table 1). From the 79 patients who received their first CI during the study period, 9 were not up-to-date with their vaccinations at the time of their candidacy assessment. Ten out of the 37 children who were receiving a second implant were not up-to-date with their vaccinations. A greater proportion of children receiving a second implant during the study period were not up-to-date with vaccinations (OR 2.35). The vaccines most commonly missed at the initial visit for first time CI recipients were HiB and/or PCV-13, whereas for those children receiving their second implant, PPV-23 was the most commonly missed vaccine (Fig. 2).

**Table 1:** Patients missing vaccinations at time of candidacy assessment and at surgery

	Patients missing vaccines at the time of assessment	Patients missing vaccines at surgery
Assessment for 1st CI		
(Normal risk patients)	9/79 (11.4%)	2/79 (2.5%)
Assessment for 2nd CI		
(High risk patients)	10/37 (27.0%)	0/37 (0%)
Total	19/116 (16.4%)	2/116 (1.7%)
Odds Ratio	OR 2.35, 95% CI:0.96–5.75, *p* = 0.06	OR 0.42, 95% CI:0.02–9.05, *p* = 0.58

### Vaccination status at the time of surgery

The Cochlear Implant Team at our institution requires all children to be up-to-date with their vaccinations prior to surgery. If the child is missing any vaccines at the time of candidacy assessment, the CI team will work closely with a vaccine specialist to develop an individualized vaccine catch-up schedule. Despite this policy, two children received a cochlear implant without being appropriately vaccinated. In both cases, the child was receiving their first CI and was missing PPV-23. All recipients who had a CI prior to the study period were up-to-date with their vaccinations at the time of the second CI surgery (Table 1).

### Vaccination status after surgery

A total of 32 children required vaccinations post-operatively. These included the two children who were operated on with missing vaccines and 30 children who were not old enough at the time of surgery to have completed their meningitis vaccination series.

**Fig. 3:**
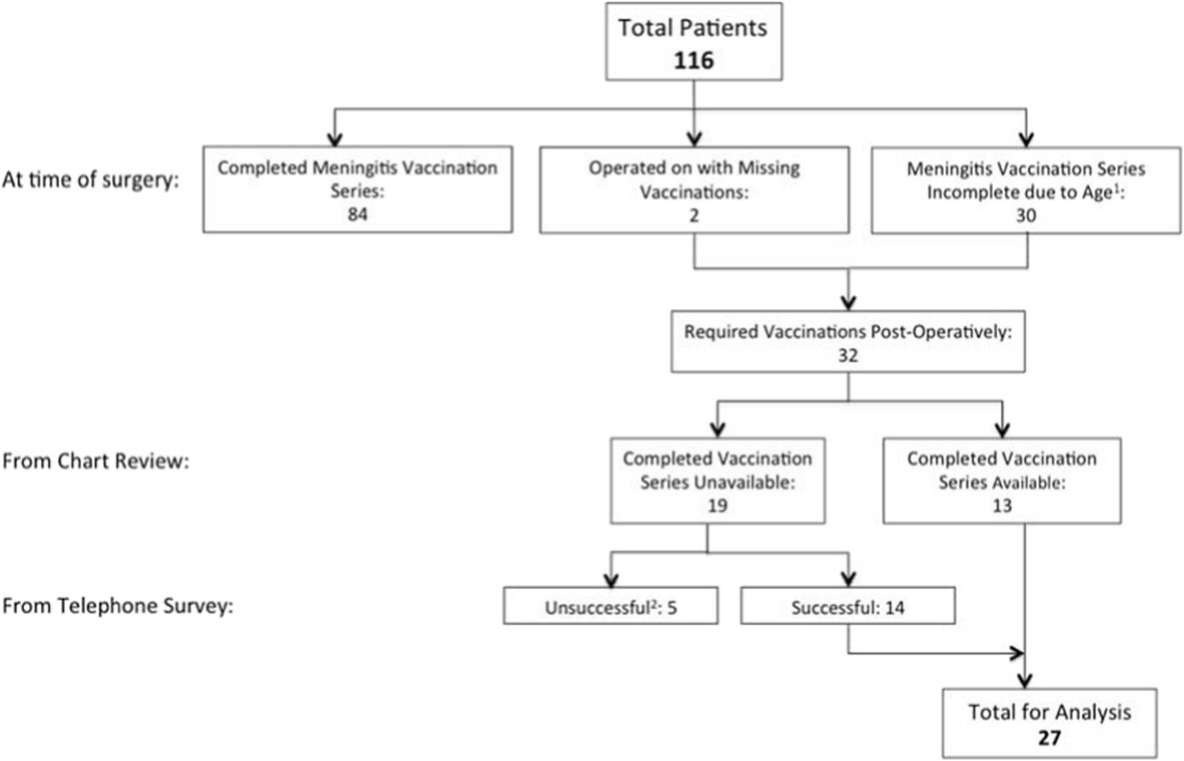
Flowchart to establish patient for post-operative immunization analysis. ^1^Children who are age-appropriately up-to-date with immunizations; however, still require additional vaccines post-operatively to complete meningitis immunization series. ^2^No consent = 1, unable to reach = 4

Completed immunization documentation was on file for 13 children, thus we attempted to contact the remaining 19 for the telephone survey. Five children did not complete the survey; 27/32 (84.4%) children were included in our post-operative immunization analysis (Fig. 3).

Out of the 27 children in our post-operative immunization analysis, 8 (29.6%) did not receive the necessary vaccination(s) after surgery. In all eight cases, the missing vaccine post-surgery was PPV-23. Four of the eight children missing PPV-23 also required additional HiB or PCV-13 vaccines, in which they received.

As previously mentioned, PPV-23 is only a requirement for high-risk children, whereas HiB and PCV-13 are part of the routine childhood immunization schedule. When asked the reason why the child missed this vaccine, the responses from the parents were similar: none of the parents were aware this vaccine was required for their child as they followed the vaccination schedule for a normal-risk child. This indicates that the main reason for non-compliance is that parents as well as Public Health were not following the high-risk schedule for cochlear implant recipients in our province.

### Comparison with previous study internal review (2002–2007)

Between 2010 and 2014, 84% (97/116) of children receiving CI were up to date with their vaccines at time of candidacy assessment with the CI team. Through individualized vaccination catch-up programs, 98% (114/116) of CI recipients were up-to-date at the time of their surgery. In comparison with the previous internal review, there was a 31% absolute increase in children being appropriately vaccinated at the time of CI surgery, from 67 to 98% (OR 28.5) (Table 2).

**Table 2:** Patients up-to-date with vaccinations at candidacy assessment and at Cochlear Implant (CI) surgery

	CI surgeries 2002-2007^a^	CI surgeries 2010-2014^b^	Comparison
Time of candidacy assessment	N/A	97/116 (84%)	–
Time of Cochlear Implant Surgery	67%	114/116 (98%)	Absolute Increase: 31% Relative Odds: 28.5

Data outlined in Table 2 compares findings from the 2008 internal review of cochlear implant surgeries between 2002 and 2007^a^ and from the current study, which reviews cochlear implant surgeries between 2010 and 2014^b^

## Discussion

Following a high-risk vaccination schedule is imperative to protect children with cochlear implants against the risk of serious, potentially life-threatening infection such as pneumococcal meningitis. Ensuring CI recipients are properly vaccinated before and after surgery is challenging due to multiple reasons. These factors, identified in our internal review, include the confusion over changes in the high-risk vaccination requirements over the years, the language barrier associated with our province’s high immigration rate, differences in the schedules among the provinces where a child might begin a schedule in one province and then move to BC. Adding to this complexity, immunizations are given in primary care settings, either by public health or family practitioners, however children are made high-risk status by the CI Team. Primary care providers need to be made aware that the patient is being considered for CI, otherwise the trigger of ‘High Risk status’ is not made.

A review of vaccination rates of CI patients at our institution in 2008 revealed that vaccination requirements were not being met and, as such, an Infectious Diseases vaccine specialist was enlisted to assist the CI Team in addressing this concern. This recent review of vaccination rates at our institution since the change was implemented indicates that pre-operative immunizations for meningitis, particularly pneumococcal meningitis, under the high-risk schedule improved significantly. There was a 31% absolute increase in the percentage of recipients who were up-to-date with vaccinations at the time of CI surgery; only 1.7% of children were operated on without having received the required vaccinations prior to surgery. Our finding highlights the success that can be achieved with the introduction of a designated specialist to monitor immunizations and provide individualized catch-up programs.

However, significant challenges remain in ensuring post-CI vaccination compliance. The 23-valent-pneumococcal-polysaccharide vaccine (PPV-23), a vaccine given specifically to patients at high risk of meningitis, is the only immunization that was reported missing in CI recipients who required immunizations post-operatively. Interestingly, children that missed PPV-23, but required other scheduled meningitis immunizations as part of the regular routine vaccines such as HiB and PCV-13 received the latter vaccinations in a timely manner. The odds of a child missing critical pneumococcal meningitis vaccinations at their visit for assessment double once the child is considered “high-risk”. This also highlights the need to follow the high-risk vaccination schedule after surgery is not well communicated to the parent or to the immunizers in primary care.

In all eight cases of missed vaccinations post-surgery, the parents reported that they were unaware this vaccine was required for their child. These parents continue to follow the vaccination schedule for a normal-risk child provided by Public Health. The parents of the children who received PPV-23 after surgery indicated that the CI team informed them of this requirement during a follow-up appointment. Only one parent reported having followed the high-risk vaccination sheet given to them during the initial consult with the CI Team. The language barrier that was previously recognized as a barrier to vaccination compliance from our internal review was no longer an active problem, as it was not identified in this study. In the initial internal review between 2002 and 2007 Cantonese and Mandarin were the two main languages that posed difficulty. Repeat calls after working day hours improved acquisition of data as English speaking parents were at home at this time. In this study, perhaps improvement was a result of the fact that in instances where the initial contact of families was with a non English speaking person, we were fortunate enough to have two of the authors who spoke Mandarin to facilitate the interaction. There are an abundant number of resources in British Columbia and specifically the Vancouver area for interpreter services and one could speculate that this has assisted in compliance as well, however our study did not address this aspect directly.

Ensuring appropriate vaccination post-surgery still remains a current issue. The key reasons for non-compliance seems to be a communication gap between the CI team, parents, family physicians and Public Health. In our province, immunizations are generally obtained at a Public Health Clinic or at a primary care clinic. During the telephone survey, we clarified to the parents which vaccination schedule their child is recommended to follow and provided them with a letter to present to the Public Health Clinic or their family physician for appropriate vaccination catch-up. However, greater measures need to be taken to close the communication gap. Solutions to bridge this gap include providing families with updated high-risk vaccination schedules post-surgery, sending reminder notifications to family members, notifying Public Health or the patient’s primary care physician regarding his/her high-risk status, or assigning a designated person or program to manage post-operative vaccinations in a similar manner to which is used in our pre-operative vaccination compliance management.

Our study revealed that immunization rates at time of surgery significantly improved after the introduction of an infectious disease vaccine specialist. Once a child was identified to be missing vaccinations during the initial candidacy assessment, individualized catch-up programs were created to ensure these children were appropriately vaccinated and would not prolong the wait time for surgery. An interesting future direction would be to assess whether this administrative change significantly impacted on the wait times for CI surgery. Our group acknowledges the difficulty of recruiting a specialist to assist in the implement of a vaccination program. Alternatively, the use of a Registered Nurse or Nurse Practitioner may exhibit similar benefits. Nonetheless, it is important to have a designated person to oversee and manage the vaccination status of a small group of high-risk individuals, such as Cochlear Implant recipients.

## Conclusion

This study showed a significant improvement was made in pre-operative vaccination rates after the introduction of a specialist. However, it is evident that a communication gap regarding which vaccination schedule to follow post-operatively continues to exist. Post-operative vaccinations are not being appropriately managed and in turn, CI patients continue to occasionally miss vaccines critical to their health. Based on the significant success in increasing pre-operative vaccination rates, one may consider creating a designated program to improve post-operative vaccinations, specifically for PPV-23. At our institution, we have ensured that all patients implanted prior to the age of 2 are seen in follow-up at 2 years of age to ensure that they are up to date for their PPV-23 vaccine.

## Abbreviations

CI: Cochlear Implant
HiB: *Haemophilus influenzae* type 2 vaccine
PCV: 13: 13-valent pneumococcal conjugate vaccine
PPV: 23: 23-valent pneumococcal polysaccharide vaccine

## References

[1] Ou Henry, Cleary Patricia, Sie Kathleen (2010). Assessing the immunization status of pediatric cochlear implant recipients using a state-maintained immunization registry. Otolaryngology-Head and Neck Surgery.

[2] Gluth MB, Driscoll CLW, Lalwani AK, Lalwani AK (2011). Cochlear implants. CURRENT Diagnosis & Treatment in Otolaryngology—Head & Neck Surgery.

[3] Reefhuis Jennita, Honein Margaret A., Whitney Cynthia G., Chamany Shadi, Mann Eric A., Biernath Krista R., Broder Karen, Manning Susan, Avashia Swati, Victor Marcia, Costa Pamela, Devine Owen, Graham Ann, Boyle Coleen (2003). Risk of Bacterial Meningitis in Children with Cochlear Implants. New England Journal of Medicine.

[4] Lalwani AK, Cohen NL (2011). Does meningitis after cochlear implantation remain a concern in 2011?. Otol Neurotol.

[5] Wei Benjamin P. C., Shepherd Robert K., Robins-Browne Roy M., Clark Graeme M., O'Leary Stephen J. (2006). Pneumococcal Meningitis Threshold Model. Otology & Neurotology.

